# Synthesis of a Flower-Like g-C_3_N_4_/ZnO Hierarchical Structure with Improved CH_4_ Sensing Properties

**DOI:** 10.3390/nano9050724

**Published:** 2019-05-10

**Authors:** Xiaojie Li, Yanwei Li, Guang Sun, Na Luo, Bo Zhang, Zhanying Zhang

**Affiliations:** 1 School of Materials Science and Engineering, Cultivating Base for Key Laboratory of Environment-Friendly Inorganic Materials in University of Henan Province, Henan Polytechnic University, Jiaozuo 454000, China; lixiaojieqq101@163.com (X.L.); liyanwei@hpu.edu.cn (Y.L.); nihaoluona@163.com (N.L.); zhb@hpu.edu.cn (B.Z.); 2 State Key Laboratory Cultivation Bases Gas Geology and Gas Control, Henan Polytechnic University, Jiaozuo 454000, China

**Keywords:** g-C_3_N_4_/ZnO, pores, heterojunction, CH_4_, gas sensor

## Abstract

In this paper, a hierarchical structure of graphite carbon nitride (g-C_3_N_4_) modified ZnO (g-C_3_N_4_/ZnO) was synthesized using a simple precipitation-calcination method. Through this method, g-C_3_N_4_ nanosheets with a controlled content were successfully decorated on the petals of flower-like ZnO. Various techniques were used to confirm the successful formation of the g-C_3_N_4_/ZnO hierarchical structure. The methane (CH_4_) sensing properties of g-C_3_N_4_/ZnO sensor were investigated. The result exhibited that after decorating ZnO with g-C_3_N_4_, the CH_4_ sensing performances of the fabricated sensor were remarkably improved. At the optimum operating temperature of 320 °C, the response of the sensor fabricated with CNZ-3 (the sample with an optimum content of g-C_3_N_4_) towards 1000 ppm CH_4_ was as high as 11.9 (R_a_/R_g_), which was about 2.2 times higher than that of the pure ZnO sensor (5.3). In addition, the CNZ-3 sensor also exhibited a fast response/recovery speed (15/28 s) and outstanding long-term stability. The enhancing CH_4_ sensing mechanism may be contributed to enlarged surface area, pore structure, and g-C_3_N_4_-ZnO n-n junction.

## 1. Introduction

Methane (CH_4_), the main constituent of natural gas, has been widely used for cooking, domestic heating, and industrial applications. However, CH_4_ is odorless, colorless, and highly flammable, which can cause some potential dangers in daily life and industrial production. Especially, CH_4_ can easily react with other gases in the air to produce dangerous explosives when its concentration in air is 5% to 15% [1,2,3]. In order to protect the safety of human life and property, developing effective method for fast and real-time detection of TEA is of great importance [4].

Over the past several decades, metal oxide semiconductor (MOS) based gas sensors have received much research interest because of their superiorities of low cost, easy fabrication, and fast response and recovery speed [5]. Now, various MOSs, such as ZnO [6], SnO_2_ [7], Fe_2_O_3_ [8], In_2_O_3_ [9], WO_3_ [10,11], TiO_2_ [12], Co_3_O_4_ [13], SnS_2_ [14], and CeO_2_ [15], have been developed as active materials for gas detection. However, as compared with the abundant research on the gas sensing properties of MOS for volatile organic compounds’ (VOCs) detection, the reports on the design and preparation of MOS nanomaterials for CH_4_ detection are relatively rare. Among the various reported MOSs, ZnO, as a typical n-type semiconductor, has been investigated as one potential gas sensing material owing to its good ability to respond to different gases [6,16]. In previous literature, ZnO was also found to be a potential material for CH_4_ detection [16]. However, due to the high chemical stability of CH_4_ molecules, most of the reported CH_4_ sensors suffered from the drawbacks of a low sensor response and long response-recover time.

Since the micro/nanostructures of MOS based on sensors have an important role in gas sensing behavior, controlling the synthesis of novel morphology and architecture of MOS nanomaterials has been a popular strategy to ameliorate gas sensing performances [17]. Recently, a three-dimensional (3D) hierarchical structure of MOS with high porosity and large specific surface area has attracted increasing interest and has been identified as a promising candidate for achieving prominent gas sensing performance [18]. In such a special structure, the high porosity and large specific surface area can endow the material with more effective channels for gas transmission and diffusion, as well as abundant active sites for gas adsorption. So, MOS with a 3D hierarchical structure always exhibits superior gas sensing properties [18,19,20]. Wang et al. synthesized flower-like WO_3_ hierarchical nanostructures through a simple two-step method of hydrothermal and calcination, which achieved a ppb-level sensitivity to NO_2_ [19]. Yang and co-works reported an ultrafast response/recovery time (0.9/1.5 s) towards trimethylamine gas through the preparation of snowflake-like α-Fe_2_O_3_ hierarchical architectures [20]. Besides the fabrication of the 3D hierarchical structure of MOS, the combination of two different nanomaterials to construct a heterostructure has also been testified to be an effective method to ameliorate gas sensing performance [21]. Many previous studies have demonstrated that heterostructured MOS materials can exhibit improved gas sensing properties as compared with their pure phase counterpart due to the synthetic effect between different sensitive materials [21,22,23,24]. Yang et al. reported the synthesis of branched SnO_2_/ZnO heterostructures and their highly enhanced ethanol gas sensing performance [22]. Hu et al. fabricated a CuO/CuFe_2_O_4_ heterostructure and found that the response value of the CuO/CuFe_2_O_4_ sensor to 10 ppm H_2_S gas was approximately 20 times higher than pure CuO [23]. Yan et al. researched the synthesis of an In_2_O_3_/ZnS heterostructure with spherical morphology via a two-step hydrothermal route. The prepared In_2_O_3_/ZnS nanocomposite showed an apparent enhancement of the ethanol sensing properties compared with pure ZnS and In_2_O_3_ [25].

G-C_3_N_4_, a novel two-dimensional material composed of carbon and nitrogen atoms, has emerged as a promising sensitizer that can improve the gas sensing properties of MOS due to its n-type semiconducting property, high specific surface area, good catalytic properties, and significant two-dimensional material characteristics [25,26,27,28,29]. Zhang et al. proved that the sensor of α-Fe_2_O_3_/g-C_3_N_4_ nanocomposites showed superior ethanol sensing performance than the pure α-Fe_2_O_3_ sensor, such as a faster response/recovery time and excellent selectivity [30]. Our group synthesized SnO_2_/g-C_3_N_4_ composites by a hydrothermal method, which showed a higher response (360) than pure SnO_2_ (230) to 500 ppm ethanol at 300 °C [28]. In previous literature, research investigating the improvement of the gas sensing properties of ZnO through combining with g-C_3_N_4_ has also been reported [29]. While most of these reports on g-C_3_N_4_/MOS are about the VOC sensing characteristics, in contrast, there are few reports focused on improving the CH_4_ properties of MOS by decorating with g-C_3_N_4_, especially to that decorating g-C_3_N_4_ on a pore sheet assembled ZnO hierarchical structure to ameliorate the CH_4_ sensing properties. 

In this work, flower-like hierarchical structures of g-C_3_N_4_/ZnO nanocomposites with different mass ratios of g-C_3_N_4_ were successfully synthesized through a simple precipitation-calcination method. Through this method, different amounts of g-C_3_N_4_ were easily decorated on the flower-like ZnO hierarchical structure that assembled with pore petals. Various techniques, including X-ray diffraction (XRD), X-ray photoelectron spectroscopy (XPS), Fourier transform infrared spectrometer (FTIR), field emission scanning electron microscope (FESEM), energy dispersive X-ray spectroscopic (EDS), and field emission transmission electron microscope (FETEM), were used to confirm the successful preparation of the g-C_3_N_4_/ZnO hierarchical structure. The (CH_4_) sensing performance of the prepared g-C_3_N_4_/ZnO was investigated and the results indicated that after decorating with g-C_3_N_4_, the ZnO sensor sensing properties towards CH_4_ were remarkably improved, especially regarding its higher response and faster response/recovery speed. 

## 2. Materials and Methods

### 2.1. Synthesis of g-C_3_N_4_

Graphite carbon nitride (g-C_3_N_4_) was prepared via a previously reported method [30,31]. Simply, a desired amount of urea was put in a horizontal tubular furnace and continuously heated in air at 250, 350, and 550 °C and maintained for 2 h, respectively. After cooling to room temperature, the last yellow powder was g-C_3_N_4_.

### 2.2. Synthesis of g-C_3_N_4_/ZnO Nanocomposite

**Figure 1:**
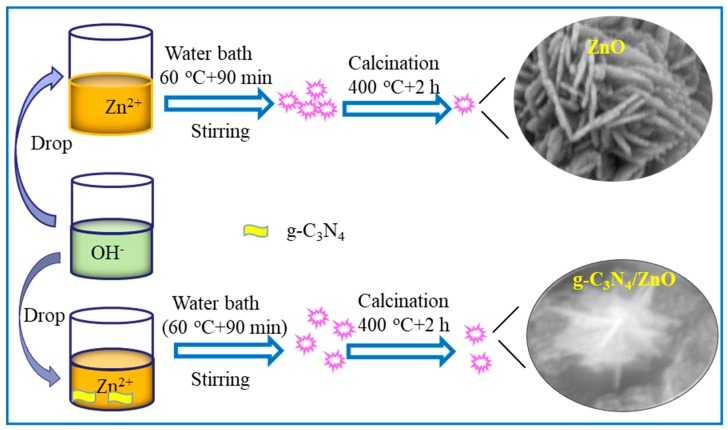
Schematic illustration for the synthesis of pure ZnO and g-C_3_N_4_/ZnO.

As shown in Figure 1, in order to synthesize the g-C_3_N_4_/ZnO nanocomposite, a desired amount of as-prepared g-C_3_N_4_ was dispersed in 20 mL of deionized water under ultrasonic irradiation for 2 h. Then, 0.571 g of Zn(NO_3_)_2_·6H_2_O was dissolved into the above suspension under vigorous stirring and 20 mL aqueous solution of NaOH (0.026 M) was added. The obtained solution was then maintained at 60 °C for 1.5 h in a water bath kettle to complete the precipitation reaction. The precipitate was obtained by centrifugation, washed with water and ethanol three times, and dried at 60 °C for 12 h. Finally, the white powder was annealed in air at 400 °C for 2 h to obtain the g-C_3_N_4_/ZnO nanocomposite. By simply adjusting the amount of g-C_3_N_4_ used, g-C_3_N_4_/ZnO nanocomposites with a g-C_3_N_4_ content of 1, 3, and 5 wt % were synthesized, which were labeled as CNZ-1, CNZ-3, and CNZ-5, respectively. For comparison, pure ZnO was also prepared under the same condition.

### 2.3. Characterization

The crystallographic structure of the samples was identified by powder X-ray diffraction (XRD, Bruker-AXS D8, Bruker, Madison, MI, USA). FESEM (QuantaTM250 FEG, FEI, Eindhoven, The Netherlands), EDS (FEI, Eindhoven, The Netherlands), and FSTEM (JEOL, Tokyo, Japan) were used to investigate the morphology of the prepared samples. Nitrogen adsorption-desorption isotherms were obtained on a Quantachrnme Autosorb-iQ sorption analyzer (Quantachrome, Boynton Beach, FL, USA). XPS (Ferkin Elmer Limited, Waltham Mass, Waltham, MA, USA) was measured to explode the analysis the elemental valence and surface. FTIR was measured on a Bruker Tensor 27 (Beijing, China).

### 2.4. Sensor Fabrication and Measurement

**Figure 2:**
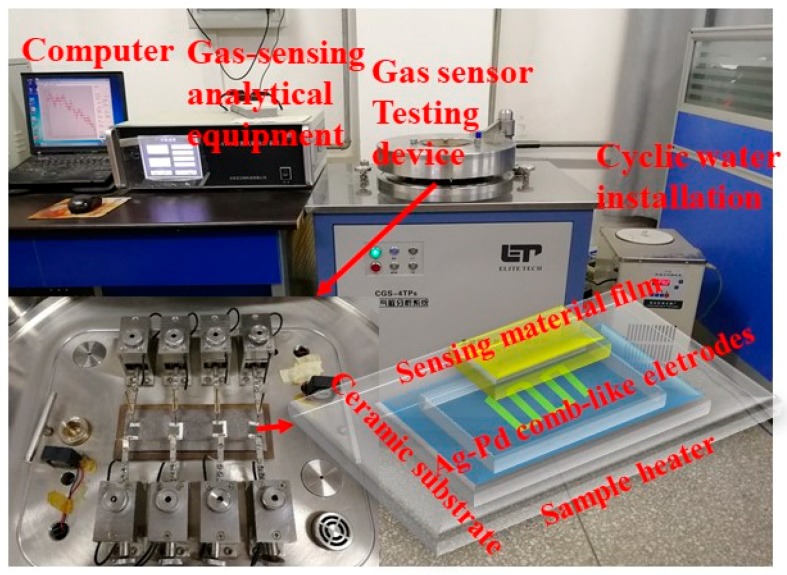
Schematic presentation of the gas sensing analysis system and 3D schematic diagram of the sensor element.

The CGS-4TPS (Beijing Elite Tech. Co., Ltd., Beijing, China), an intelligent gas sensing analysis system, was used to test the gas sensing properties, and the complete device is exhibited in Figure 2. The structure of the gas sensor is clearly shown in Figure 2, consisting of four parts, a sample heater (direct contact heating device), ceramic substrate (Al_2_O_3_), Ag-Pd comb-like electrodes (resistance signal of the transmission device), and sensing materials. In order to reduce the error of the test, the gas sensor was used strictly in accordance with the quantitative requirements during the manufacturing process. Firstly, 10 mg of prepared powers of CNZ was mixed with two drops of deionized water to form a homogenous paste and the paste was brush-coated on the surface of a ceramic substrate. Then, the substrates were dried at 60 °C for 24 h to obtain the sensor. Finally, the fabricated sensors were aged at 200 °C for 4 h to improve their stability. During testing, a static volumetric method was adopted to obtain different concentrations of CH_4_ in the closed test chamber. A certain amount of CH_4_ was injected into the test chamber (1.8 L) by a syringe and the relative humidity was around 35% in this measurement.

## 3. Results and Discussion

### 3.1. Morphology and Structure of the ZnO and g-C_3_N_4_/ZnO Nanocomposites

**Figure 3:**
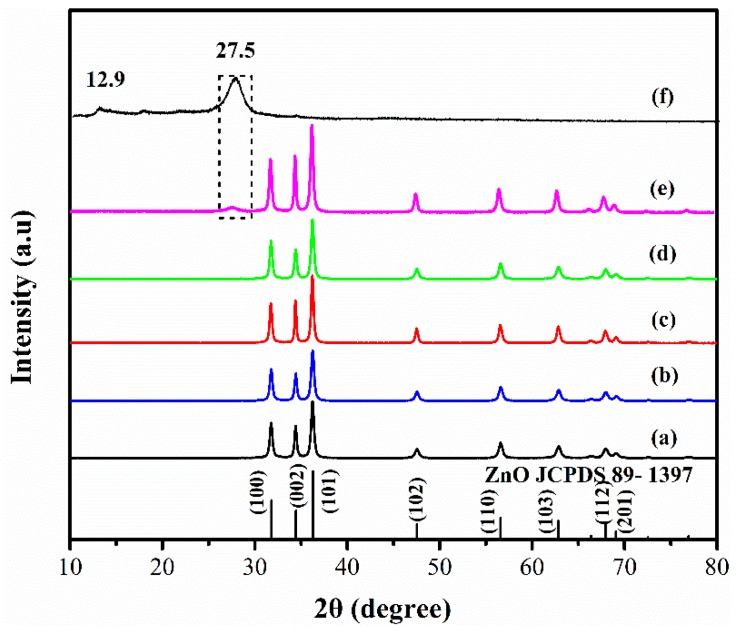
XRD patterns of the prepared samples: (**a**) pure ZnO, (**b**) CNZ-1, (**c**) CNZ-3, (**d**) CNZ-5, and (**e**) g-C_3_N_4_/ZnO composites with a g-C_3_N_4_ content of 20 wt %, (**f**) g-C_3_N_4_.

The compositions of the sample were first analyzed by XRD. Figure 3a shows the pure ZnO sample XRD pattern. All diffraction peaks matched well with the standard date of hexagonal ZnO (JCPDS: no. 89-1397), indicating the purity of the ZnO sample. Diffraction peaks of the prepared g-C_3_N_4_ are displayed in Figure 3f. In this Figure, two diffraction peaks located at 12.9° and 27.5° are clearly observed, which can be assigned to the (100) and (002) planes of g-C_3_N_4_, respectively. The XRD patterns of the prepared g-C_3_N_4_/ZnO composites are shown in Figure 3b–d, in which the characteristic peaks arising from ZnO can be clearly observed, but no obvious diffraction peaks of g-C_3_N_4_ are detected. The absence of the peaks from g-C_3_N_4_ can be attributed to the relatively low content of g-C_3_N_4_ in the composite and the results are similar with the studies of Cao [28]. In addition, the g-C_3_N_4_/ZnO composites with a g-C_3_N_4_ content of 20 wt % were prepared and the XRD are shown in Figure 3e. To be sure, the g-C_3_N_4_/ZnO composites were successfully synthesized in this work.

**Figure 4:**
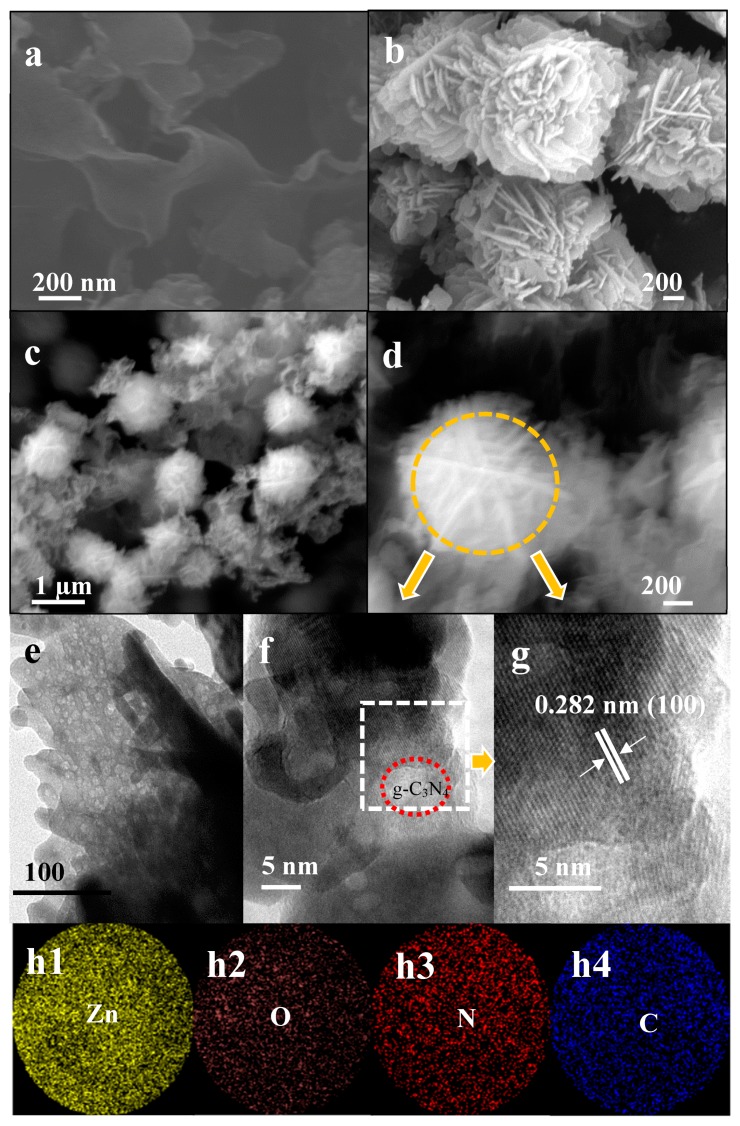
FESEM images of g-C_3_N_4_ (**a**), pure ZnO (**b**), and CNZ-3 (**c**,**d**), (**e**,**f**) TEM and (**g**) HRTEM image of CNZ-3, (**h1**,**h2**,**h3**,**h4**) elemental mapping of Zn, O, N, and C (CNZ-3).

From Figure 4a, one can observe that g-C_3_N_4_ nanosheets were successfully prepared, and in Figure 4b, it can be seen that many 3D hierarchical microstructures with a flower-like morphology formed in the pure ZnO sample. Similar flower-like microstructures were also observed in the g-C_3_N_4_/ZnO nanocomposites, as shown in Figure 4c,d, indicating that the introduction of g-C_3_N_4_ nanosheets has almost no influence on the morphology of the products. Meanwhile, the TEM of ZnO petals are exhibited in Figure 4e. A large number of randomly dispersed nanopores with a size of about 1 to 5 nm were embedded on the petals, resulting in the formation pore petals in the flower-like architecture [32]. The existence of these nanopores on the ZnO petals can not only enlarge the specific surface area of the composite sensing materials, but also promote gas diffusion and transmission in the materials, both of which are considered to be helpful for improving the gas sensing properties. Detailed structural information of the prepared flower-like CNZ-3 was revealed by TEM observation. Figure 4f gives a typical TEM image recorded from CNZ-3, in which the g-C_3_N_4_/ZnO composites are clearly observed, consistent with the results given by FESEM observation. Figure 4g presents an enlarged TEM image of the observed flower-like composites and the lattice fringe spacing was measured to be 0.282 nm, in good agreement with the (100) plane of ZnO (JCPDS: no. 89-1397). Around the crystalline ZnO sheets, some g-C_3_N_4_ phase was also observed. From the high magnification FESEM images of CNZ-3 shown in Figure 4f,g, we can clearly see that the g-C_3_N_4_ nanosheets closely attached to the ZnO petals, suggesting that (n)ZnO-(n)g-C_3_N_4_ junctions can be created in the composite material.

In order to confirm the successful introduction of g-C_3_N_4_ into the flower-like ZnO, EDS measurement was performed on the prepared composite sample. Figure 4(h1,h2,h3,h4) show the EDS mappings recorded from an isolated flower observed in Figure 4c. Besides the Zn and O, the signals from C and N were also observed, suggesting that the observed flower was composed of g-C_3_N_4_ and ZnO.

**Figure 5:**
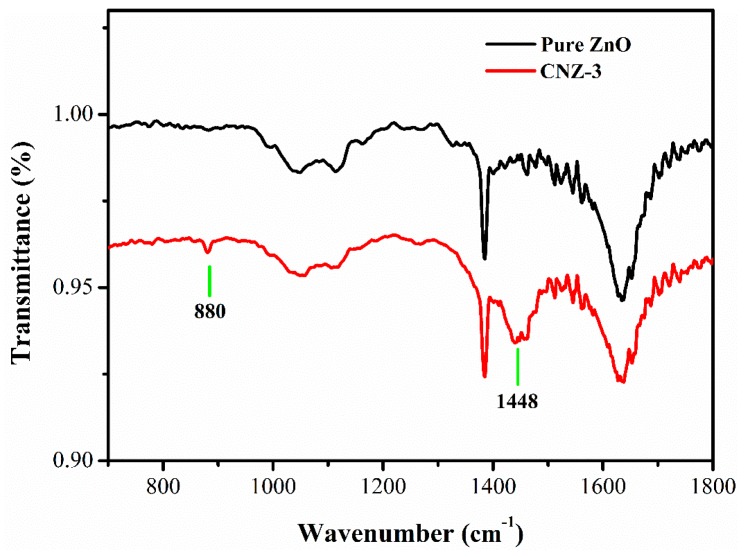
FTIR spectra of the pure ZnO and CNZ-3.

The FTIR spectra of the prepared pure ZnO and CNZ-3 were recorded. As shown in Figure 5, compared with the pure ZnO sample, two additional peaks located at 1448 cm^−1^ and 880 cm^−1^ were observed in the CNZ-3 sample, which can be attributed to the typical stretching vibration of CN heterocycles [33] and the characteristic breathing mode of the triazine units of g-C_3_N_4_, respectively.

**Figure 6:**
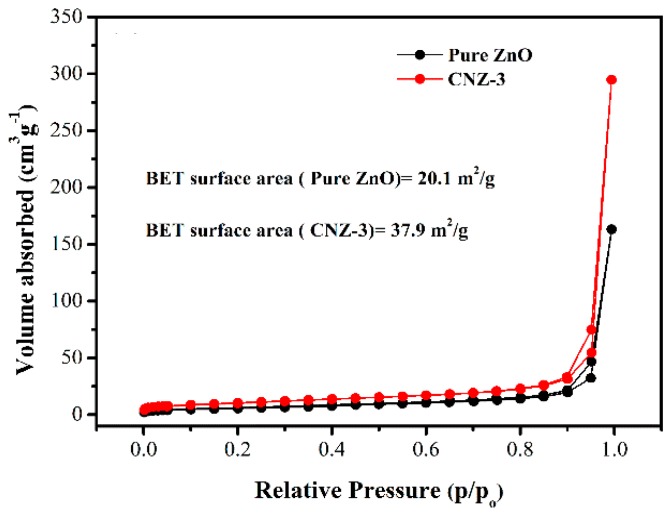
N_2_ adsorption-desorption isotherms.

The specific surface area and pore size distribution of the ZnO and CNZ-3 samples were investigated by N_2_-sorption measurements and the results are shown in Figure 6. In Figure 6, typical type-IV isotherm curves are shown. The BET surface areas were calculated to be 37.9 m^2^/g and 20.1 m^2^/g for CNZ-3 and ZnO, respectively. The larger specific surface area of CNZ-3 could be attributed to the introduction of g-C_3_N_4_.

**Figure 7:**
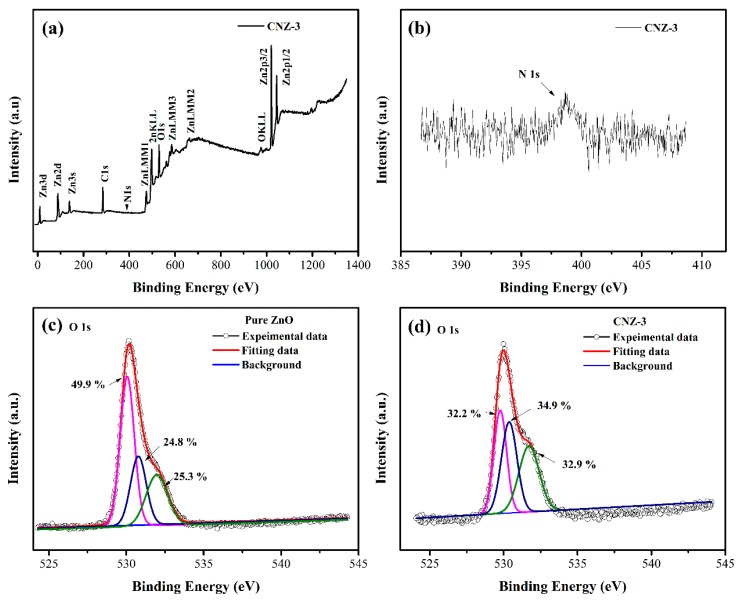
XPS spectra of the prepared sample: (**a**) full survey scan spectrum of CNZ-3, (**b**) high resolution of N 1s spectrum of CNZ-3, and O1s spectra of (**c**) pure ZnO and (**d**) CNZ-3.

The chemical bond state of the prepared samples was investigated with XPS. Figure 7a shows the full survey scan spectrum recorded from CNZ-3, in which four elements, including Zn, O, C, and N, were identified, further proving the successful introduction of g-C_3_N_4_ into the flower-like ZnO. A weak N 1s peak at about 498.4 eV was observed in the high resolution spectra of N1s (Figure 7b), similar with the results reported by Kouki et al. [34]. The low intensity of the N 1s peak could be attributed to the relatively low content of g-C_3_N_4_. Figure 7c and d show the O 1s spectra of the pure ZnO and CNZ, respectively. Three kinds of oxygen species were obtained by fitting the O 1s peaks, including lattice oxygen (O_L_), oxygen vacancy (O_V_), and chemisorbed oxygen (O_C_), and the relative percentages of the O_V_ and O_C_ components were 32.9% and 34.9% for CNZ-3, and 25.3 and 24.8% for ZnO, respectively.

### 3.2. Gas Sensor Fabrication and Analysis

**Figure 8:**
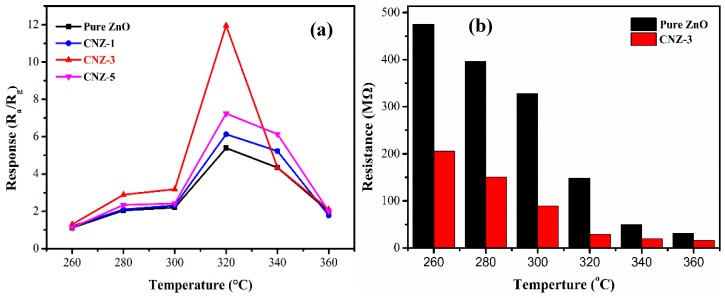
(**a**) The temperature-dependent response of the sensors to 1000 ppm CH_4_; (**b**) the resistances in air (Ra) of the pure ZnO and CNZ-3 sensors at different operating temperatures.

The decoration of g-C_3_N_4_ nanosheets on the porous petals-assembled flower-like ZnO was expected to bring improved gas sensing properties, thus the CH_4_ sensing performance of the g-C_3_N_4_/ZnO nanocomposites was investigated to explore their possible application in CH_4_ detection. During gas sensing tests, the pure ZnO sample was used as a reference. Considering that the operating temperature influences MOS sensors, the temperature-dependent response was first tested to obtain the optimum operating temperature. Figure 8a shows the response values of the sensors to 1000 ppm CH_4_ at different operating temperatures (ranging from 260 to 360 °C). All sensors showed the highest response value at 320 °C. Such a result means that the gas adsorption and desorption on the surface of sensitive materials is balanced at this temperature. While, in the relatively low temperature range (260–320 °C), the gas adsorption speed will increase with the increase of the operating temperature, resulting in a continuously increased response. Conversely, when the temperature is over 320 °C, the speed of gas desorption will exceed that of gas adsorption, which will lead to a decreased response with further increases of the temperature from 320 to 360 °C. So, 320 °C is determined as the optimum operating temperature. Meanwhile, the optimal g-C_3_N_4_ content of 3 wt% in the present composite system is exhibited.

**Figure 9:**
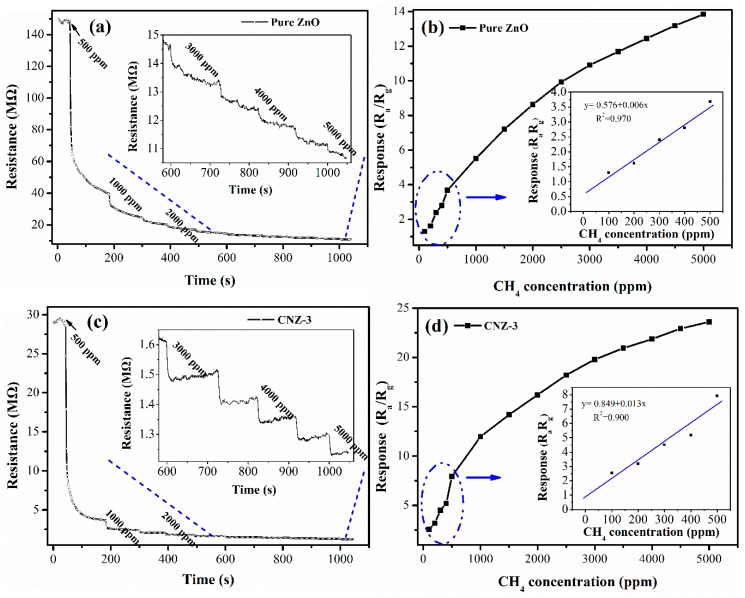
Dynamic electrical responses of the (**a**) ZnO and (**c**) CNZ-3 sensors with the CH_4_ concentration increasing from 500 ppm to 5000 ppm at 320 °C; the concentration-dependent response curves of (**b**) ZnO and (**d**) CNZ-3.

The dynamic response–recover curves as well as the concentration-dependent response of the sensors based on ZnO and CNZ-3 are presented in Figure 9. As shown in Figure 9a,c, once exposed to 500 ppm CH_4_, both sensors gave a fast decrease in resistance, exhibiting characteristic n-type semiconducting properties. With a further increase of the CH_4_ concentration, the resistances of the two sensors decreased correspondingly. While, as compared with the pure ZnO sensor, the CNZ-3 sensor gave clear and regular response steps to different concentrations of CH_4_ (insets in Figure 9a,c), revealing its better ability to respond to CH_4_. The response variation depending on the CH_4_ concentration of the ZnO and CNZ-3 sensors is shown in Figure 9b,d, respectively. With successive increases of the CH_4_ concentration, both sensors achieved an obvious enhancement of the response and a good response linearity within 100 to 500 ppm. While, the response values of the CNZ-3 sensor to various concentrations of CH_4_ were always higher than the ZnO sensor, demonstrating the sensitization effect of g-C_3_N_4_ on ZnO during the sensing of CH_4_. Based on the fitting lines shown in the insets of Figure 9b,d, the detection limit (DL) of the sensors was estimated, which reference the literature [35]. The sensor noise was extracted using 20 points and the slope was obtained, as shown in Figure 9b [35]. Using the above equation, the D_L_ of the pure ZnO and CNZ-3 sensors were calculated to be 10.2 ppm and 0.4 ppm, respectively. The lower D_L_ value of CNZ-3 implies its better ability to detect low concentrations of CH_4_.

**Figure 10:**
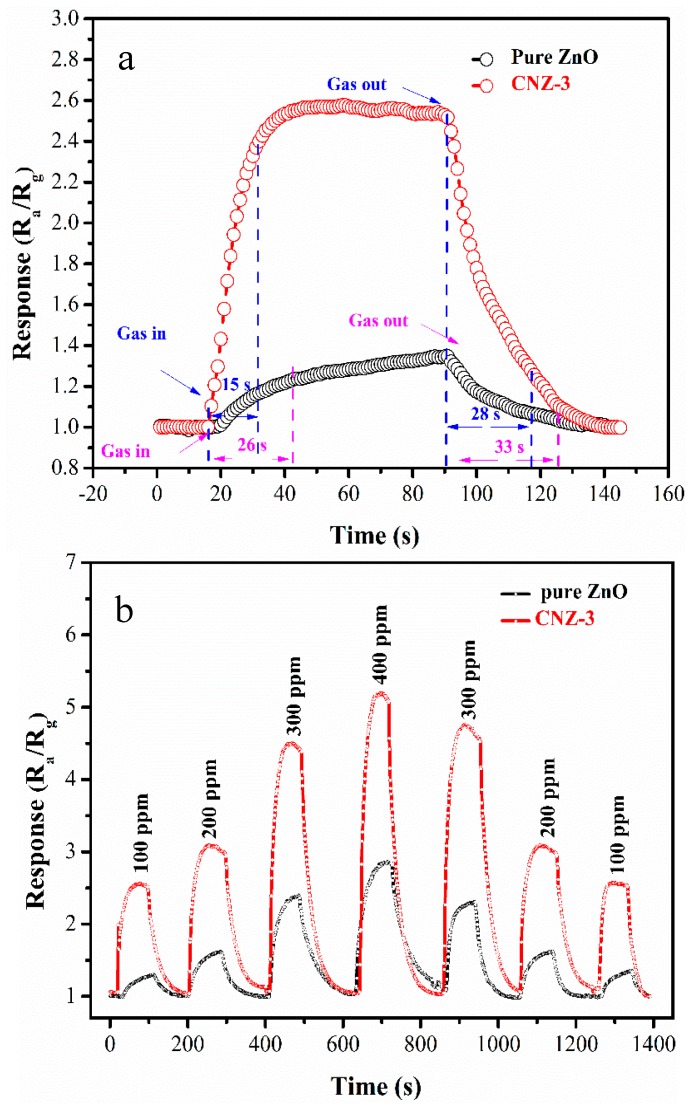
(**a**) Transient response-recover curves of the sensors towards 100 ppm CH_4_ at 320 °C; (**b**) dynamic response of the sensors to different concentrations of CH_4_ at 320 °C.

Figure 10a shows the sensors’ transient response-recovery curves to 100 ppm CH_4_. Based on this figure, the measured response/recovery (T_res/_T_rec_) times of the ZnO and CNZ-3 sensors are 14/18 s and 26/33 s. The dynamic response of the sensors to four different concentrations of CH_4_ at 320 °C are shown in Figure 10b. The response amplitudes of the sensors increased correspondingly with the CH_4_ concentration increasing (from 100 ppm to 400 ppm). Importantly, the CNZ-3 sensor can give similar response amplitudes to the same concentration of CH_4_, demonstrating its good repeatability.

**Figure 11:**
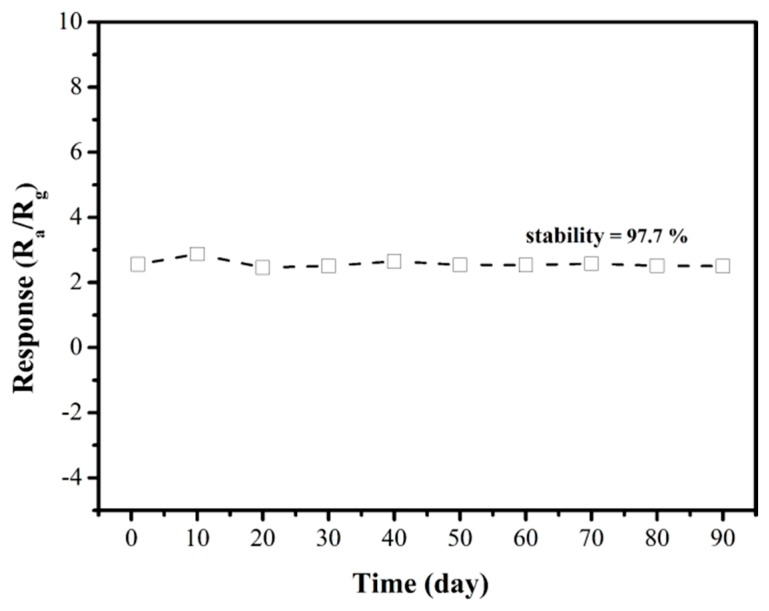
Long-term stability of the CNZ-3 sensor towards 500 ppm CH_4_ at 320 °C.

In addition, the long-term stability of the sensors based on CNZ-3 was also tested, as shown in Figure 11. It can be seen that the CNZ-3 sensor showed a small fluctuation in its response in the first month perhaps due to the sensor aging and a disappearance of unstable adsorption sites. While, after 90 days, the sensor could still maintain 97.7% of the initial response value (stability = 97.7%), which indicates that the sensor has excellent long-term stability. In order to further evaluate the quality of the CNZ-3 sensor, its CH_4_ sensing properties weree compared with different kinds of sensitive materials, as shown in Table 1. Clearly, the present g-C_3_N_4_/ZnO sensor showed a remarkable improvement in sensor response and response/recovery speed.

### 3.3. Gas Sensing Mechanism

**Figure 12:**
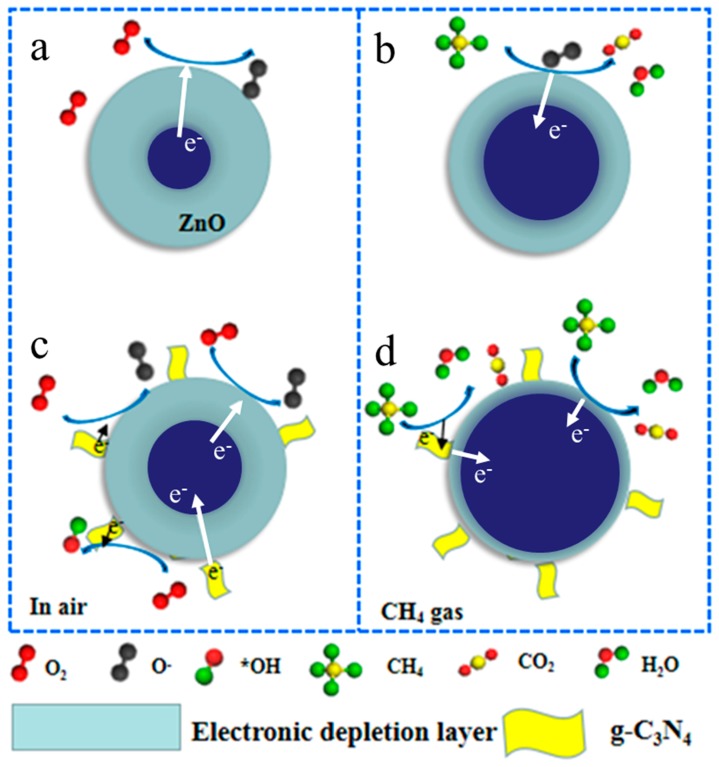
Schematic illustration of CH4 sensing mechanism of pure ZnO (**a**,**b**) and g-C_3_N_4_/ZnO (**c**,**d**).

The gas sensing mechanism of ZnO can be explained by the varied thickness of the electronic depletion layer (EDL) formed on the surface of ZnO when the senor is switched from air to reducing gas atmospheres [22,28,31]. In our experiment, when the sensor was exposed to air, oxygen molecules adsorbed on the surface of ZnO and then captured electrons from the conduction band of ZnO to form various types of chemisorbed oxygen anions, such as O^−^, O^2−^, and O_2_^−^, resulting in the formation of a thick EDL, as shown in Figure 12a. In this case, a relatively higher sensor resistance (R_a_) can be obtained because of the low conductivity of the EDL. When CH_4_ was injected into the test chamber, the redox reaction between CH_4_ molecules and chemisorbed oxygen anions will occur on the ZnO surface. Meanwhile, the electrons will be released back to ZnO, which are captured by oxygen anions. So, a lower sensor resistance (R_g_) will be exhibited due to the decrease of the EDL thickness, as shown in Figure 12b.

In our experiments, it was found that after decorating ZnO with g-C_3_N_4_, the response of the sensor to CH_4_ was remarkably improved. The improved CH_4_ sensing behaviors regarding CNZ-3 can be mainly attributed to three factors. Firstly, the results of the N_2_ sorption analysis indicated that after decorating ZnO with g-C_3_N_4_, the specific surface area of the composite material was remarkably increased. In fact, the specific surface area of CNZ-3 was found to be 37.9 m^2^/g, which was much larger than that of pure ZnO (20.1 m^2^/g). The larger specific surface area of CNZ-3 means that during the gas sensing process, more active sites can be provided for gas adsorption, thus leading to a higher response. In addition, the result of the O 1s XPS spectra (Figure 7) shows that the relative percentage of the O_c_ component in CNZ-3 (32.9%) is more than that of pure ZnO (25.3%). The higher O_c_ value in CNZ-3 indicates that the decoration of g-C_3_N_4_ on ZnO can produce more O_c_ anions on the surface of the composite material, resulting in, during the gas sensing process, more chemisorbed oxygen anions provided to react with CH_4_ molecules.

Another important reason for the improved CH_4_ response of the composite nanomaterial sensors should be the n-n heterojunction formed between g-C_3_N_4_ and ZnO. As observed by HRSEM and HRTEM (Figure 4), (n)ZnO-(n)g-C_3_N_4_ junctions were formed. The n-n junctions may bring two positive effects. First, the different lattice parameters between ZnO and g-C_3_N_4_, leading to a great number of defects, formed between ZnO and g-C_3_N_4_, which became potential active sites, thus causing a higher response of the composite sensor as compared with the pure ZnO sensor. Form the result given by XPS analysis, it can be found that the O_V_ value in CNZ-3 (34.9%) was obviously higher than that in pure ZnO (24.8%), revealing that due to the formation of the g-C_3_N_4_-ZnO n-n heterojunction, more defects (oxygen vacancy) were created in the CNZ-3 sample. Secondly, because the Fermi levels of g-C_3_N_4_ are higher than ZnO [45,46], at the interface of g-C_3_N_4_ and ZnO, electrons will flow from g-C_3_N_4_ to ZnO until their Fermi levels reache equalization. Thus, once the CNZ-3 sensor is exposed to the CH_4_ vapor, the electrons that were trapped by chemisorbed oxygen anions will be released back to ZnO, and additional electrons coming from g-C_3_N_4_ can also be poured into ZnO through the n-n heterojunction, leading to a much thinner EDL (Figure 12d). Thus, a higher CH_4_ response can be obtained on the CNZ-3 sensor.

In addition, the good catalytic property of g-C_3_N_4_ was also considered to be helpful for the improved CH_4_ response of the g-C_3_N_4_/ZnO nanocomposites. In the air atmosphere, g-C_3_N_4_ can promote oxygen molecules to form functional groups, such as hydroxyl (_*_OH), which can facilitate the oxidation of CH_4_ molecules [47,48].

## 4. Conclusions 

In summary, a g-C_3_N_4_ decorated ZnO hierarchical structure was synthesized via a facile precipitation-calcination method, through which g-C_3_N_4_ nanosheets with a controlled content were successfully decorated on the ZnO hierarchical structure assembled with pore petals. The results displayed that after decorating with g-C_3_N_4_, the ZnO sensor CH_4_ sensing properties were remarkably enhanced. The response of the sensor fabricated with CNZ-3 towards 1000 ppm CH_4_ was as high as 11.9, which was about 2.2 times higher than that of the pure ZnO sensor (5.3) at the optimum operating temperature of 320 °C. Furthermore, the CNZ-3 sensor also exhibited a fast response/recovery (15/28 s) speed and good long-term stability, demonstrating its potential application in CH_4_ detection. The present research demonstrates that decorating ZnO with two-dimensional g-C_3_N_4_ to construct n-n junctions is a practical method to enhance the gas sensing performance of ZnO. In future work, we will consider other porous nanomaterials [49] with g-C_3_N_4_ to improve the CH_4_ gas sensor performance. 

## Figures and Tables

**Table 1:** Comparison of the CH_4_ sensing performance of different reports.

Sensitive Materials	Temp. (°C)	S (R_a_/R_g_)	CH_4_ Con. (ppm)	T_res_/T_rec_ (s)
SnO_2_/Au [36]	400	0.82	3000	--
Fe-doped SnO_2_ [37]	350	0.67	1000	--
Ca-doped Pt-catalyzed SnO_2_ [38]	400	17	5000	--
Mesopores SnO_2_ [39]	600	0.6	4000	--
Pd-Nanopores SnO_2_ [40]	600	20	6000	--
MoO_3_ [41]	500	10	500	--
Pd–Al_2_O_3_/SnO_2_ [42]	450	10	4000	--
Pd-doped SnO_2_/rGO [43]	Rt	10%^*^	14,000	300/420
WO_3_/SnO_2_ [44]	110	2.9	500	--
G-C_3_N_4_/ZnO This work	320	2.56	100	15/28

^*^$\left(\;\frac{\mathrm{Ra}-\mathrm{Rg}}{\mathrm{Ra}}\times 100\;\%=S\;\%\right)$.
